# Screening HIV associated neurocognitive disorders using international HIV dementia scale: closing the gap through an educational intervention for healthcare workers

**DOI:** 10.11604/pamj.2023.44.17.33844

**Published:** 2023-01-09

**Authors:** Jane Kasozi Namagga, Grace Nambozi, Godfrey Zari Rukundo, Vallence Niyonzima, Vincent Batwala

**Affiliations:** 1 Department of Nursing, Faculty of Medicine, Mbarara University of Science and Technology, Mbarara, Uganda,; 2 Department of Psychiatry, Faculty of Medicine, Mbarara University of Science and Technology, Mbarara, Uganda,; 3 Department of Community Health, Faculty of Medicine, Mbarara University of Science and Technology, Mbarara, Uganda

**Keywords:** HIV-associated neurocognitive dementia, international HIV dementia scale, knowledge, educational intervention, healthcare workers

## Abstract

**Introduction:**

the study assessed the effect of an educational intervention on healthcare workers´ knowledge regarding the use of the International HIV Dementia Scale (IHDS) in screening HIV-associated neurocognitive disorder (HAND) at The AIDS Support Organization (TASO) centres in Uganda.

**Methods:**

we recruited healthcare workers in southwestern and central Uganda. Data were collected by a questionnaire, cleaned, and analyzed using means and standard deviations. A paired t-test assessed mean knowledge score differences pre-and post-intervention. We used One-Way ANOVA for mean score differences between sites and cadres. Statistical significance was taken at p ≤ 0.05 and 95% confidence interval. Prevalence of HAND for clients screened during educational intervention was computed.

**Results:**

mean age was 36.38 years (SD = 7.80) and mean years of experience 8.92 (SD = 6.52). A paired t-test showed that pre-intervention mean score (Mean= 20.38, SD 2.94) was statistically different from post-intervention mean score (Mean=22.24, SD 2.15) at t (36) = - 4.933, p > 0.001). One-way ANOVA showed counselors were statistically different from clinical officers´ pre-intervention (Mean difference 4.432 (95% CI: 0.1- 8.85, p= 0.049) and post-intervention (Mean difference 3.364 (95% CI: 0.07 - 6.65, p= 0.042) respectively. There was no difference in mean knowledge scores between sites pre-intervention (F (4, 32) = 0.827, p = 0.518) and post-intervention (F (4, 32) = 1.299, p = 0.291). Of the 500 clients screened, 72.2% were positive for HAND.

**Conclusion:**

the educational intervention improved healthcare workers´ knowledge regarding screening HAND using IHDS at TASO centres in Southwestern and Central Uganda.

## Introduction

Globally, the survival rate associated with Human Immunodeficiency Virus (HIV) infection has improved dramatically since the introduction of antiretroviral therapy (ART) in the mid-1990s [1]. ART has transformed HIV and AIDS once a deadly infection into a chronic disease linked with a near-normal lifespan [2]. Regardless of the improved life expectancies, people living with HIV (PLWH) are at risk of developing HIV- associated neurocognitive disorders (HAND) [3]. This may perhaps be due to the persistent progressive immunosuppression before initiation of ART, and continuing viral replication within the brain notwithstanding the achievement of viral suppression [4,5]. HAND is a spectrum of neurocognitive dysfunctions ranging from subtle neuropsychological impairments to severely incapacitating HIV-associated dementia [6] that causes significant declines in cognitive, motor, and/or behavioral domains [3]. This encompasses asymptomatic neurocognitive impairment (ANI), mild neurocognitive disorder (MND), and HIV-associated dementia (HAD) depending on the functional impairment [7]. Although some studies have reported significant reduction in HAD in the ART era, less severe forms of HAND persist with a prevalence of 20-50% [3,8], memory and executive function impairment being more evident [3,6,9]. This hinders individuals´ ability to perform activities of daily living and adherence to their essential medications that adversely affect their quality of life [3]. HAND remains a major cause of 15 to 55% of morbidity and mortality worldwide irrespective of better healthcare systems [3].

Hence, this necessitates strategic efforts to aid with early diagnosis to decrease the direct and indirect effects of HIV replication within the central nervous system (CNS) [10]. This could be achieved by effective agents, good adherence, tolerance to ART, and close monitoring [9]. Therefore, specific measures and interventions need to be established as more than 50% of PLWH have poor insight into their cognitive deficits [11]. Screening for HAND in routine HIV care is one of the important strategy in recognizing changes in cognitive functioning that allow early interventions [12]. Although healthcare workers (HCWs) are at the forefront of HIV care to provide holistic and equitable care, they may not routinely screen for HAND. In their collegial working relationship, the knowledge and skills regarding screening HAND in routine HIV care are not known [13]. A healthcare worker in this study is one who offers HIV care and includes; medical officers, clinical officers, nurses and counselors. Guidelines for HIV care entail HIV prevention, counseling and testing, linkage to care and follow-up, treatment, and retention in care and psychosocial support to PLWH and their families [14]. Although the Ugandan HIV guidelines added routine screening for depression to improve HIV care delivery [15], little consideration has been given to HAND screening.

This remains an under-appreciated challenge in the overall management of HIV despite HAND being a hidden epidemic [9]. An earlier study by Valcour and colleagues [16] reported that screening for HAND in all PLWH is crucial for early detection of complications. However, the best approaches of screening have not been determined for clinical contexts especially in resource-limited settings, including Uganda [17]. This gap leaves the majority of PLWH not screened for HAND in routine HIV care [18]. Whereas screening was not a common practice in the past due to limited knowledge of cognitive impairment associated with HIV infection, the trend is changing today [19]. There is an increasing understanding of the consequences of HAND that has led to the utilization of simple validated screening tools such as the International HIV Dementia Scale (IHDS [20].

The IHDS was first developed by Power, Seines [21] and, since 2005, many studies have demonstrated its efficiency in a variety of populations [22-25]. The tool was initially validated in the United States and Uganda and showed sensitivities and specificities of 80% and 57%, and 80% and 55% respectively using a cut-off score of <10 (scores 0-12). The maximum score of 12 is indicative of better performance [25]. It has also been used successfully in South Africa [26], Ethiopia [27], Nigeria [28], Malawi [29], Botswana [30] and India [31]. The IHDS has the advantage of not requiring any unique instrument except a timer or wristwatch. This is a cheap and valuable tool that is particularly recommended for screening in outpatient clinics [9,25]. The efficiency of this tool has been found to offer the best practice and helps to organize access to further neuropsychological diagnoses for better health outcomes [24]. Therefore, this makes it an attractive option for use in resource-limited settings like Uganda particularly in TASO centres.

The best practice recommends that a patient´s neurocognitive profile be assessed at least before the initiation of ART or within 6 months of diagnosis, using a sensitive screening tool [32]. Similarly, a group of authors agreed that screening for HAND should occur every 6 to 12 months in higher-risk patients, every 12 to 24 months in lower-risk patients and instantly in case of any clinical deterioration [10]. The formal assessment of neurocognitive disorders is a complete neuropsychological assessment done by a trained neuropsychologist [33,34]. However, such comprehensive assessments are not available in daily clinical practice, which results in silent suffering for PLWH with HAND. Optimal assessments require objective evaluation of everyday function which is the cornerstone of identifying HAND which is a mismatch in the study setting. Yet, it is clinically important to recognize HIV- related morbidity and mortality [35] because a missed diagnosis can impede on health outcomes of PLWH [36]. Equally, early recognition can have profound clinical implications today and in the future [37]. Therefore, screening for HAND is an essential approach that can optimize brain health, improve well-being and avoid a social burden to the family, community and the country at large. There is a paucity of literature on the knowledge of HCWs regarding the use of IHDS in screening HAND. This study assessed the effect of an educational intervention on healthcare workers´ knowledge regarding the use of IHDS in screening HAND at TASO centres in central and southwestern Uganda.

## Methods

**Study design and setting**: an educational intervention was conducted between April 2020 and September 2021 at five (5) centres of The AIDS Support Organization (TASO) in central and southwestern Uganda. TASO founded in 1987, is the indigenous, oldest, and the largest organization providing HIV/AIDS care and support services in Uganda and Sub-Saharan Africa [38]. The organization has the widest HIV/AIDS service delivery network in Uganda and directly complements the efforts of the Ministry of Health. TASO offers a comprehensive care package including medical care, psychosocial support, and sensitization about adherence to medication, especially ART. We recruited participants from Mulago, Masaka, Mbarara, Entebbe and Rukungiri centres to provide some diversity. The aforementioned, TASO Mulago situated in Kampala district in central Uganda started in 1987. This was the first centre to be established in Kampala, and being the capital city, it hosts a cross-section of people, transient and migrant populations typical of a developing city. By 2018, TASO Mulago had served and improved the lives of 7,901 clients 5,733 (female) and 2,168 (male), their families, and communities. TASO Masaka, the second centre to be established in 1988 is located within Masaka Hospital premises. It is along the transport corridor to Rwanda and Tanzania border in Mutukula. Masaka is known for neighboring Rakai District where the first cases of AIDS were identified in 1980s. By 2018, TASO Masaka had served and improved the lives of 37,000 clients 25,900 (70% female) and 11,100 (30% male), their families and communities.

Subsequently, TASO Mbarara was opened in 1989. This is a fully-fledged and semi-autonomous centre adjacent to Mbarara Regional Referral Hospital along the transport corridor to Rwanda. The population include traders passing through from one border to another and pastoralists who are transient by nature. By 2018, TASO Mbarara had served and improved the lives of 6, 399 clients 4,146 (female) and 2,253 (male), their families and communities. TASO Entebbe within Entebbe Municipal Council started working in 1991. Entebbe being urbanized have an ever-growing and transient population including traders, fisher folk, uniformed personnel, and also a first and last stop for international tourists. By 2018, TASO Entebbe had served and improved the lives of 6,292 clients 4165 (female) and 2,124 (male), their families, and communities. TASO Rukungiri opened in 2004, is located along Ishaka Road about 600 metres from Municipal Town Council. Most of the population live in hard to reach areas with hills and valleys which makes accessibility to medical services challenging. By 2018, TASO Rukungiri had served and improved the lives of 7,200 clients 4,728 (female) and 2, 472 (male), their families and communities. Although these centers are located in the urban district towns/cities, they have extensive service delivery networks up to grassroots communities. They provide a range of services to clients from within a radius of 75 kilometers. The study sites were purposively selected because they are variations in culture, level of urbanization, development and social ramifications. With 25 years of experience in HIV/AIDS care and treatment, the five TASO centres provided excellent study sites to assess the effect of an education intervention regarding screening HAND using IHDS by healthcare workers (HCWs).

**Study population**: the study population was healthcare workers working in the five TASO centres in central and southwestern Uganda. There were 90 in total including; medical officers, nurses, clinical officers, and counselors that were offering routine HIV care services. All participants selected were aged 18 years and above, and were eligible to participate in the study. Potential participants were excluded if they were interns on placement because they had not yet gained experience and part-time healthcare workers to minimize confounding relations.

**Sample size estimation**: using a list of healthcare workers obtained from the medical coordinators of the study sites, a total of 67 participants met the inclusion criteria. The sample size of 67 healthcare workers was not calculated since the population was small. We recruited all healthcare workers who were eligible for the pre-post educational intervention. However, due to workload pressures, 30 participants dropped out because of other competing activities such as; ongoing studies, community engagements, and off-site workshops that needed constant time. Only 37 participants successfully completed all three phases of the educational intervention. We thus were unable to include the full number of participants in the study as originally envisioned.

**Data collection tool**: we used a questionnaire to elicit responses from the participants. The development of the questionnaire was informed by a review of published literature on screening HAND. We came up with 25 unique questions which formed the preliminary version of the questionnaire. The questionnaire was evaluated following a method described by Armstrong and colleagues [39], that involves obtaining judgmental evidence. The content validity index was computed for each item (I-CVI) and content validity ratio (CVR). Five health care professionals with experience in HIV care and research rated the relevance, clarity, and essentiality of the items in the questionnaire. They were given a critical appraisal sheet to independently rate: 1) the relevance of each question (how important the question is), 2) the clarity of each question (how clear the wording is), and 3) the essentiality of each question (how necessary the question is). Relevancy was assessed using a 4-point Likert scale where: 1=not relevant, 2=somewhat relevant, 3=quite relevant and 4 =very relevant [40]. Clarity was on a 3-point Likert scale where 1=not clear, 2= items that need some revision, and 3=very clear [41]. Essentiality was evaluated on a 3-point Likert scale where 1=not essential, 2=useful, but not essential, and 3 = essential [39,41]. After evaluation, the I-CVI of each item ranged from 0.8 to 1.00. All the twenty-five items were retained. The Content Validity Ratio (CVR) measured the essentiality of an item [41], which varies between 1 and -1 [41]. It is stated that a higher score indicates greater agreement among the evaluators [39]. The CVR ranged from 0.2 to 1 and considered all the twenty-five items essential. Equally, the clarity of each item was calculated on a 3-point Likert Scale where 1 = not clear, 2 = somewhat clear, and 3 = very clear. Average clarity scores for individual items ranged from 2.4 to 3.00. The overall clarity score was 2.74 indicating that all the items were generally clear. Therefore all the questions in the questionnaire were retained and used for the data collection.

**Data collection procedure**: we collected data between April 2020 and September 2021 using the pre and post-educational intervention questionnaires. The study took longer than expected because of the C-19 pandemic lockdown. We assessed the pre-educational status of participants´ knowledge regarding the use of IHDS in screening HAND and any post-educational changes. The content of the training was designed to increase participants´ knowledge on the use of IHDS in screening HAND. The questionnaire format involved scoring statements (correct or not correct). We standardized the intervention by ensuring the same procedures, similar questionnaires, and trained research assistants in the entire process of data collection in all the five study sites.

This was done in three phases:

**Phases 1**: this involved administration of a pre-intervention questionnaire which was followed by hands-on training on the use of the International HIV Dementia Scale (IHDS) in screening HAND for one week. The IHDS is a brief and rapid screening tool that evaluates memory recall, motor and psychomotor speed [25]. The tool adopted from Sacktor and colleagues [25], was initially validated in Uganda and the United States using a cut- off of ≤10. The IHDS consists of three assessments: (a) timed finger tapping (FT) for motor speed; (b) timed alternating hand sequence (AHS) for psychomotor speed (c) and a 4-word recall (4WR) after two minutes for memory registration. The maximum possible score is 12 as per standard protocol [25]. Participants who scored ≤10 were screened as having a risk of HAND. The tool was recommended for use in this study because it is easy to administer once trained and requires no sophisticated instrumentation other than a timer or a watch with a second-hand ticker. The training was conducted in English, a language understood and spoken by all participants.

**Phase 2**: upon completion of phase 1, participants worked with the research team in their daily clinical practice as they offered routine HIV care services. For the acquisition of knowledge to accurately interpret the tool, participants were required to screen at least 10 clients every day. This was a hands-on practice that also identified day-to-day challenges related to the usage of the tool in the care. The research team provided support through guidance, coaching, mentoring, debriefing, and feedback on a daily basis specifically for two weeks in this phase and for the entire period of the intervention. This helped participants gain knowledge, competency, and confidence as they applied the tool and interpreted it correctly.

**Phase 3**: this phase proceeded with continuous support, guidance, and monitoring from the research team as participants practiced independently while offering routine HIV care. Throughout this time, the details of each client screened were recorded for more interpretation. Clients that screened positive for HAND were given appointments for additional assessment before being referred to a psychiatrist for comprehensive assessment and management. Four weeks after completing the intervention, participants were subjected to a post- intervention a questionnaire using similar questions to evaluate the effect of the educational intervention.

**Data management and analyses**: the questionnaires were checked for completeness, data were coded, entered in Microsoft excel, and cleaned for missing values before analysis. The cleaned data were analyzed using Statistical Package for Social Scientists (SPSS) version 23.0. Continuous variables were analyzed using means and standard deviations while frequencies were used for categorical variables. We used a paired t-test to assess for differences in the pre-and post-intervention mean scores. One Way ANOVA was used to assess for differences in mean scores between the study sites and different cadres. The level of statistical significance was taken at a p-value ≤0.05 and a 95% confidence interval. Additionally, the prevalence of HAND for clients that were screened by participants during the study period was computed.

**Ethical considerations**: ethical approval was sought and received from Research Ethics Committee in Mbarara University of Science and Technology (No. 27/10-16) and Uganda National Council for Science and Technology (No. HS2194). Administrative clearance was granted by TASO Institutional Review Committee at the National level. Informed consent was gained from each participant, who self-identified as interested in taking part. Participants´ confidentiality was maintained by study identification numbers. The informed consent forms that contained participants´ names were stored separately. All databases were password protected.

## Results

**Socio-demographic analysis**: in total, 37 healthcare workers (medical officers, clinical officers, nurses, and counsellors) participated in the educational intervention. The mean age was 36.38 years (SD = 7.80) while the mean years of experience were 8.92 (SD = 6.52). The study involved almost an equal number of males (n=18, 49%) and females (n=19, 51%). A vast majority (62.2%) of the participants were married, 44.5% were diploma holders while 37.8% of the participants were registered nurses (Table 1).

**Table 1:** sociodemographic characteristics

Variable	Mean	SD
**Age**	36.38	7.80
**Years of experience**	8.92	6.52
**Variable**	**Frequency (n)**	**Percentage (%)**
**Gender**		
Male	18	48.6
Female	19	51.4
**Marital status**		
Single	12	32.4
Married	23	62.2
Divorced	2	5.4
**Qualification**		
Certificate	5	13.6
Diploma	17	45.9
Degree	15	40.5
**Cadre**		
Enrolled Nurse	5	13.6
Registered Nurse	14	37.8
Clinical officer	4	10.8
Medical officer	3	8.1
Counselor	11	29.7

**Descriptive analysis**: outcomes assessed were changes in the scores before and after the educational intervention. Participants´ knowledge regarding the use of IHDS in screening HAND significantly improved. Each participant was required to indicate whether the statement was correct or incorrect. There was an overall increase in knowledge in 22 out of 25 variables after the educational intervention. However, there was a decrease in knowledge about the item that assessed whether the asymptomatic neurocognitive impairment is a disorder that does not affect activities of daily living (Table 2). Using one way ANOVA, post hoc tests indicated that counsellors were statistically different from clinical officers pre- intervention (Mean difference 4.432 (95% CI: 0.1-8.85, p= 0.049) and post- intervention (Mean difference 3.364 (95% CI: 0.07-6.65, p= 0.042) respectively. A paired t test showed that the overall pre- intervention mean score (Mean= 20.38, SD 2.94) was statistically different from the overall post- intervention mean score (Mean=22.24, SD 2.15) at t (36) = - 4.933, p > 0.001 (Table 3). With one way ANOVA, post hoc tests indicated that there was no statistically significant difference in the mean knowledge scores between the different sites pre-intervention (F(4, 32) = 0.827, p = 0.518) and post-intervention (F (4, 32) = 1.299, p = 0.291) (Table 4). During the intervention, participants screened a total of 500 clients using IHDS. Out of these 386 (77.2%) screened positive for HAND. Masaka had the highest (90%) while Mulago had the lowest (67%) number of clients that screened positive (Table 5).

**Table 2:** participant knowledge regarding usage of IHDS in screening for HAND

SN	STATEMENT	Pre-intervention		Post-intervention		
		Correct n(%)	Incorrect n (%)	Correct n(%)	Incorrect n(%)	% Change
1	The human immunodeficiency virus infects cells of the immune system, impairing their function leading to immune deficiency	37(100)	0(0)	37(100)	0(0)	0.0
2	The immune system is considered deficient when it can no longer fulfill its role of fighting infection and disease within the body	37(100)	0(0)	37(100)	0(0)	0.0
3	CD4+ cells or T-helper cells are a type cells that play an important role in the immune system. They suppress or regulate immune responses in the body	34(91.9)	3(8.1)	36(97.3)	1(2.7)	5.4
4	Infections that are associated with severe immunodeficiency are known as opportunistic infections because they take advantage of a weakened immune system	35(94.6)	2(5.4)	37(100)	0(0)	5.4
5	The brain is the second most frequently affected organ by HIV virus and is associated with morbidity	30(81.1)	7(18.9)	35(94.6)	2(5.4)	13.5
6	Neurocognitive disorders are one of the major complications among people living with HIV	33(89.2)	4(10.8)	35(94.6)	2(5.4)	5.4
7	A collection of neurocognitive disorders are referred to as HIV-associated neurocognitive disorders (HAND)	31(83.8)	6 (16.2)	34(91.9)	3 (8.1)	8.1
8	HAND causes a decline in brain function, movement skills, as well as shifts in behavior and moods	31(83.8)	6(16.2)	35(94.6)	2 (5.4)	10.8
9	Asymptomatic neurocognitive impairment is a disorder that does not affect activities of daily living	29(78.4)	8 (21.6)	26(70.3)	11 (29.7)	-8.1
10	Mild neurocognitive disorder mildly affects activities of daily living	28(75.7)	9(24.3)	33(89.2)	4 (10.8)	13.5
11	HIV-associated Dementia is a disorder that severely affects activities of daily	28(75.7)	9 (24.3)	29(78.4)	8 (21.6)	2.7
12	An undetectable viral load is not a risk factor for developing HAND in people living with HIV	14(37.8)	23 (62.2)	25(67.6)	12 (32.4)	29.8
13	A high CD4+ count is a risk factor for developing HAND in people living with HIV	29(78.4)	8 (21.6)	35(94.6)	2 (5.4)	16.2
14	International HIV Dementia Scale is one of the tools used to screen for HAND	32(86.5)	5 (13.5)	35(94.6)	2 (5.4)	8.1
15	The IHDS tool has three parameters for assessing neurocognitive cognitive disorders (Psychomotor, motor and memory recall)	35(94.6)	2(5.4)	36(97.3)	1 (2.7)	2.7
16	A person suffering from HIV associated dementia displays reduced concentration	7(18.9)	30 (81.1)	36(97.3)	1 (2.7)	78.4
17	A person suffering from HIV associated dementia is not able to maintain focus on life and complete important tasks	7(18.9)	30 (81.1)	33(89.2)	4 (10.8)	70.3
18	A person suffering from HIV associated dementia has decreased reading abilities and less interest in the surrounding environment	29(78.4)	8 (21.6)	32(86.5)	5 (13.5)	8.1
19	People suffering from HIV associated dementia have reduced memory and need reminders	2(5.4)	35 (94.6)	35(94.6)	2 (5.4)	89.2
20	A person suffering from HIV associated dementia has speech changes, slowing and sometimes difficulty in finding words	29(78.4)	8 (21.6	35(94.6)	2 (5.4)	16.2
21	A person suffering from HIV associated dementia has difficulty in making decisions	29(78.4)	8 (21.6)	34(91.1)	3 (8.1)	12.7
22	A person suffering from HIV associated dementia suffers from imbalance and body weaknesses	23(62.2)	14 (37.8)	32(86.5)	5 (13.5)	24.3
23	Personality behavioral changes are rare in people suffering from HIV associated dementia	9(24.3)	28 (75.7)	29(78.4)	8 (21.6)	54.1
24	Sleep disturbances, generally excessive day time sleepiness are common in people suffering from HIV associated dementia	30(81.1)	7 (18.9)	32(86.5)	5 (13.5)	5.4
25	Not all people living with HIV should be screened for HAND	10(27)	27 (73)	32(86.5)	5 (13.5)	59.5

IHDS: International HIV Dementia Scale tool; HAND: HIV-associated neurocognitive disorder

**Table 3:** mean knowledge scores according to cadre

Cadre	Pre-intervention Mean (SD)	Post-intervention Mean (SD)	Difference in Mean
Enrolled nurse	21.20(3.63)	21.80(2.68)	0.60
Registered Nurse	21.14 (1.88)	22.79(1.42)	1.65
Clinical officer	22.25(.50)	24.00(.82)	1.75
Medical officer	22.33(2.08)	24.00(1.00)	1.67
Counselor	17.82 (3.06)	20.64(2.29)	2.82
Overall	20.38(2.94)	22.24(2.15)	1.86

**Table 4:** mean knowledge scores according to site

Site	Pre-intervention Mean (SD)	Post- intervention Mean (SD)	Difference in Mean
Rukungiri	19.62 (4.81)	21.25(2.96)	1.63
Mbarara	19.67(2.16)	22.33(2.42)	2.66
Masaka	21.10(1.524)	22.80(1.398)	1.7
Mulago	19.75(3.059)	21.63(2.200)	1.88
Entebbe	22.00(1.581)	23.60(.548)	1.6
Overall	20.38 (2.938)	22.24(2.153)	1.86

**Table 5:** screening for HAND using IHDS during the study period

Site	IHDS SCORE		TOTAL n (%)
	HAND (≤10) n(%)	No HAND (> 10) n (%)	
**Masaka**	45(90)	5(10)	50(100)
**Rukungiri**	90(75)	30(25)	120(100)
**Entebbe**	114(84.4)	21(15.6)	135(100)
**Mbarara**	78(72.9)	29(27.1)	107(100)
**Mulago**	59(67)	29(33)	88(100)
**TOTAL**	386(77.2)	114(22.8)	500(100)

HAND: HIV-associated neurocognitive disorder; IHDS: International HIV Dementia scale

## Discussion

We aimed at assessing the effect of an educational intervention using IHDS in screening HAND by healthcare workers at TASO centres in central and southwestern Uganda. The mean knowledge scores for both pre and post-intervention were computed for different cadres and study sites. While counselors were statistically different from clinical officers, there was no statistically significant difference among study sites probably due to the fact that the standard of care is similar across TASO centres. Participants were also trained to use IHDS in screening HAND during routine HIV care and a total of 500 clients were screened. Out of these 386 were found positive for HAND giving a prevalence of 77.2%. Findings are discussed in the context of routine HIV care offered at TASO centres where usage of IHDS in screening HAND is rare. Surprisingly, studies of educational interventions on the use of IHDS are limited in the literature. Our discussion was based on studies that focused on the effect of education interventions irrespective of the field. Based on this fact, we did an educational intervention for healthcare workers to assess its effect. Our results clearly show an increase in participants´ knowledge regarding the usage of IHDS in screening HAND. These findings may be explained by the fact that participants were provided guidance, mentoring, and debriefing in real-time throughout the study for seven weeks. Our findings coincided with what has been found in other studies concerning the effect of an educational intervention on participants´ knowledge. For example, Opadeyi and colleagues [42] in Nigeria reported that an educational intervention had a positive impact on the knowledge and practice of pharmacovigilance among healthcare professionals. Congruently, another study on the effect of educational interventions among healthcare providers in Jordan demonstrated a knowledge increase [43]. A similar pattern of results was obtained in Iraq [44]. This reflects that different educational interventions may be comparable across settings regardless of the field.

On the other hand, our study indicated that counselors were statistically different from clinical officers in the pre and post- educational interventions. The findings are substantial given the fact that the two cadres have varying training details in their preparation. The clinical officers undertake comprehensive training in physical assessment of clients while the counselors are more didactic in their approach and are mostly trained in psycho-social support. Contrarily, there was no statistically significant difference between the different study sites pre and post- educational intervention. This could probably have been due to the education level, years of experience that were similar across the study sites, and the same standard of care being offered. These results also indicate the commonalities in the scope of practice and training that explicitly expose a clear education system for healthcare workers in Uganda. With the majority of PLWH being asymptomatic, screening for HAND remain extremely limited in clinical settings [9,22]. Knowledge acquisition in screening HAND using IHDS is significant in mitigating the health effects of HIV/AIDS and its complications [45]. Thus, routine screening could partake a positive drive in targeting the unrecognized asymptomatic disease [46].

During the intervention, when participants independently screened for HAND, a prevalence of 77.2% was registered. The high prevalence could have been an overestimate due to other comorbid conditions that affect PLWH. Another reason could be due to the wrong reading of results on the first encounter when the scores are inappropriately interpreted. Devoting more time to practicing using IHDS may improve healthcare workers' knowledge in screening for HAND. Gisslén *et al*.[46] asserted that HAND diagnosis is only if the cognitive impairment cannot be explained by other comorbidities, which may be difficult to identify. Although screening for mental disorders is recommended by HIV guidelines [47], there is no consensus on tools to use [48,49]. It is fundamental to improve the quality of care for PLWH and reduce the burden in resource - limited settings like Uganda. Therefore, the approach of using IHDS is useful as repeated screening may contribute to better health outcomes.

**Strengths and weaknesses of the study**: the main strength of the study is that the educational intervention created awareness and skepticism toward HAND screening in PLWH at TASO centres. However, there were some weaknesses that could be considered. First, the use of pre and post-design was weak in evaluating the educational intervention without comparison to a control group. Second, the data collection time period was relatively short, pre-post evaluations without following -up with healthcare workers and assessing their knowledge over a long period of time may have affected the study outcome. Third, small sample size was used in this study hence, results may not necessarily be generalizable to other HIV care centres. Fourth, the high dropout rate due to other competing activities may equally have affected the results. Nonetheless, the current study provides a valuable contribution, because healthcare workers can use this information for timely detection and appropriate mitigation of HAND in routine HIV care.

## Conclusion

On the basis of our findings, it can be concluded that educational intervention was effective in improving healthcare workers´ knowledge regarding the use of IHDS in screening HAND at TASO centres in southwestern and central Uganda. This may also inform the development of new policies and interventions to help healthcare workers improve the efficacy of their HIV care services and deliver better quality care to PLWH. The impact of the organizational culture, and ministry of health policies could be important aspects in sustaining the effectiveness of such educational interventions. **Recommendation**: involving other services such as psychiatry support when managing this segment of the population could be beneficial. We also recommend a qualitative study exploring the experience of healthcare workers regarding the use of IHDS in screening HAND in routine HIV care.

### What is known about this topic

- HAND is a major cause of morbidity and mortality worldwide even in its mildest form among PLWH;
- Screening for HAND remains extremely limited in clinical settings with no consensus on screening tools yet the majority of PLWH are asymptomatic;
- This gap leaves the majority of PLWH not screened for HAND in routine HIV care which affects their quality of life.

### What this study adds

- The study created awareness and improved the knowledge of healthcare workers on HAND screening using IHDS among PLWH;
- In addition, the current study provides valuable contributions to healthcare workers; this information may be used for early detection and management of HAND in the routine HIV care.
